# Relationship of Pelvic Positional Change with Leg Length and Offset Measurement in Experimental Total Hip Arthroplasty

**DOI:** 10.1111/os.13728

**Published:** 2023-04-24

**Authors:** Naoya Kikuchi, Haruo Kawamura, Tomofumi Nishino, Hajime Mishima

**Affiliations:** ^1^ Department of Orthopaedic Surgery, Faculty of Medicine University of Tsukuba 1‐1‐1 Tennodai, Tsukuba Ibaraki 305‐8575 Japan; ^2^ Department of Orthopaedic Surgery North Prefecture Medical Center, Takahagi Kyodo Hospital 1006‐9 Agehocho, Kamitetsuna, Takahagi Ibaraki 318‐0004 Japan

**Keywords:** Leg Length, Offset, Pelvic position, Total hip arthroplasty

## Abstract

**Objective:**

During total hip arthroplasty (THA), both pelvic and femur positions affect leg length (LL) and offset (OS) measurements because LL and OS calipers depend on the fixed reference points on the pelvis and femur, respectively. However, LL and OS measurement errors because of pelvic positional changes have not been described. This study aimed to clarify the effects of pelvic positional changes on LL and OS measurements in relation to the pelvic reference using a THA simulator.

**Methods:**

We developed an experimental THA simulator using Sawbones models of the hemipelvis and femur that facilitated modification of the obliquity, tilt, and rotation of the pelvis. Using an LL and OS caliper, LL and OS measurement errors due to pelvic positional changes were determined with the femoral position fixed. Measurements were performed from two pelvic reference positions: the iliac tubercle (P1) and the top of the iliac crest intersecting the line of the femoral long axis (P2).

**Results:**

Concerning pelvic obliquity, the total error of LL was 25.0 mm in P1 and 26.5 mm in P2, while the total error of OS was 13.0 mm in P1 and 10.9 mm in P2. For pelvic tilt, the total error of LL was 9.0 mm in P1 and 3.8 mm in P2, while the total error of OS was 0.5 mm in P1 and 1.0 mm in P2. Regarding pelvic rotation, the total error of LL was 13.8 mm in P1 and 3.2 mm in P2, while the total error of OS was 3.8 mm in P1 and 4.0 mm in P2.

**Conclusions:**

Pelvic positional changes alter LL and OS measurements. The acceptable range (error <2 mm) on LL and OS measurement errors of pelvic obliquity was only 2°, regardless of the pelvic reference position. The pelvic reference position should be at the top of the iliac crest intersecting the line of the long axis of the femur because of a small LL measurement error with pelvic tilt and rotation.

## Graphical abstract

Pelvic positional changes alter leg length and offset measurements. Pelvic obliquity had an enormous effect on leg length and offset measurement errors.

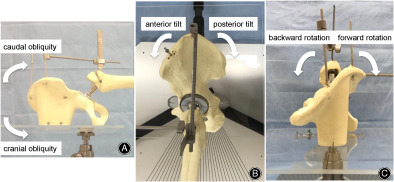

## Introduction

Total hip arthroplasty (THA) is a standard treatment modality for end‐stage hip arthritis. To achieve an optimal functional result after THA, it is necessary to properly adjust the leg length (LL) and offset (OS) within the hip. LL inequality after THA is a major cause of patient dissatisfaction due to unsatisfactory outcomes, such as limping, knee and back pain, early prosthesis loosening and revision surgery, and litigation.^1^, ^2^, ^3^, ^4^, ^5^, ^6^, ^7^, ^8^ Furthermore, improper OS adjustment also causes issues such as hip joint instability, decreased range of motion, and gait alteration.^7^, ^8^, ^9^, ^10^, ^11^

**Fig. 1:**
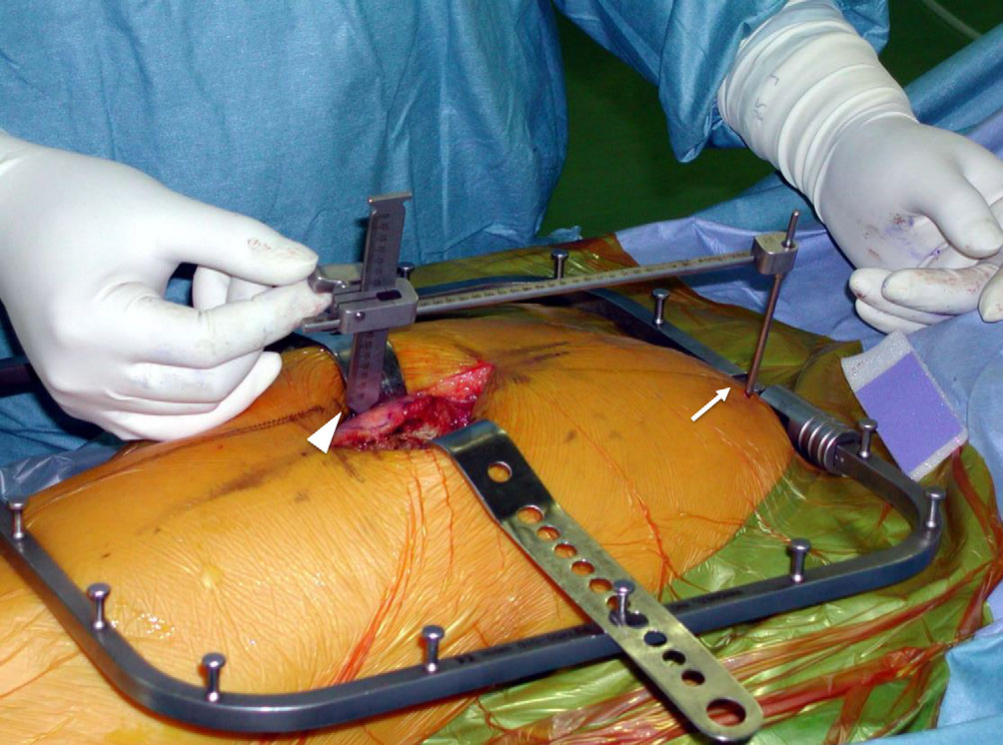
The leg length and offset measurements during total hip arthroplasty. The LOMI is connected to a pin inserted into the iliac tubercle (arrow). The lateral prominence of the greater trochanter is marked with electrical cautery (arrowhead). The leg length and global offset are measured using LOMI. LOMI: leg length and offset measurement instrument

To obtain appropriate LL and OS, preoperative planning is necessary to determine the extent to which LL and OS will change before and after surgery, and it is desirable to be able to confirm these changes intraoperatively. The LL and OS measurement instrument (LOMI; Smith & Nephew, Memphis, TN, USA) is a unique device that can detect changes in LL and OS before hip dislocation and after trial component or final implant insertion.^12^ In clinical practice, a pin is inserted into the iliac tubercle through a stab incision for pelvic reference. Regarding the femoral reference, the lateral prominence of the greater trochanter is marked with electrical cautery. Then, the LOMI is set on a stopper of the iliac pin, and the LL and OS are measured before hip dislocation and after the trial component or final implant insertion (Fig. 1).

Given that the fixed points are distant from the center of rotation of the hip, the positions of the femur and pelvis affect LL and OS measurements.^6^ To measure the LL and OS accurately, it is essential to maintain the pelvis and femur in precisely the same position between measurements. The effects of femur position on LL and OS measurements have already been investigated in different experimental settings.^13^, ^14^ The pelvic position is known to change during THA surgery, especially with pelvic tilt and obliquity,^15^, ^16^, ^17^, ^18^ resulting in variation in acetabular component orientation.^19^ Similar to the femur, managing positional changes in the pelvis improves the accuracy of LL and OS measurements, but no studies have quantitatively examined the measurement error of LL and OS due to pelvic positional change. Moreover, the iliac tubercle, which is situated anterior to the hip rotation center, is known to be a non‐ideal pelvic reference for accurate LL measurement.^14^ Therefore, the purpose of this study was: (i) to clarify the effect of pelvic positional changes on measurement errors of the LL and OS; and (ii) examine the effect of two pelvic reference positions on measurement errors of the LL and OS using a THA simulator.

## Methods

### Experimental THA Simulator

**Fig. 2:**
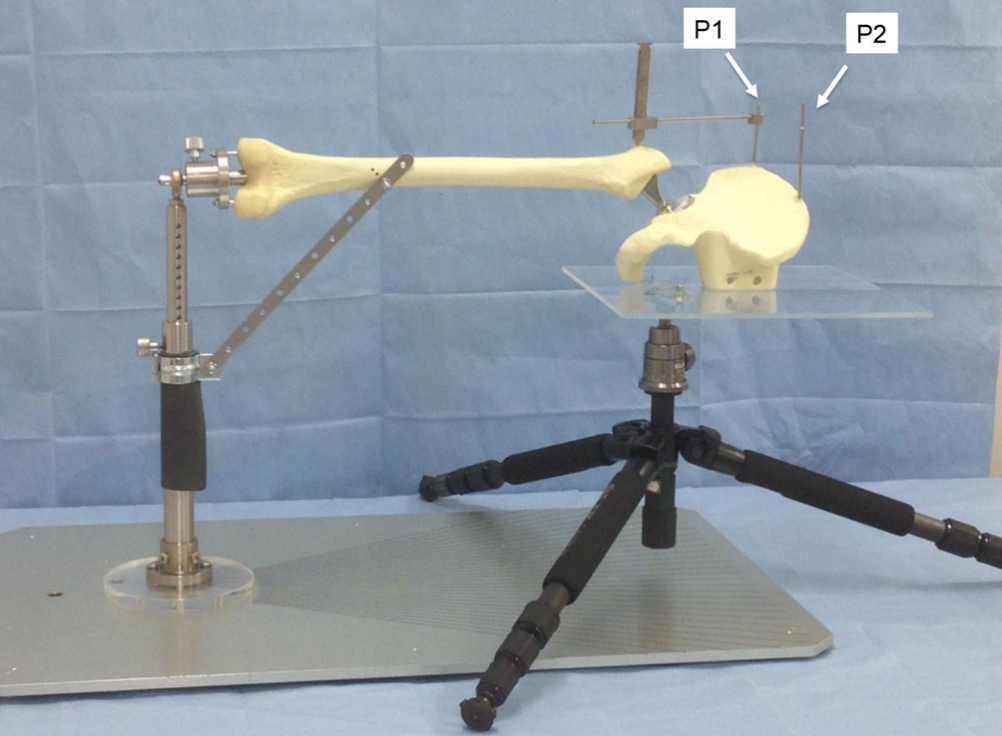
General view of a custom‐made experimental total hip arthroplasty device. The position of the pelvis can be changed using a free‐camera platform and a tripod. The femur is fixed to a baseboard. P1: Iliac tubercle. P2: The top of the iliac crest intersecting the line of the long axis of the femur when the pelvis and the femur are in a neutral position. In this photograph, the leg length and offset measurement instrument is set on P1 with the pelvis and the femur are set in neutral position

A custom‐made experimental device was developed to simulate THA surgery in the lateral decubitus position (Figs 1 and 2). In our previous study, the accuracy and repeatability of the LOMI on the THA simulator were found to be within 0.3 and 0.9 mm, respectively.^14^ A 56‐mm acetabular metal shell and an appropriate polyethylene liner (Reflection SP3 and XLPE, Smith & Nephew) were firmly fixed to a Sawbone model of the hemipelvis (model 1304, Sawbones, Vashon Island, WA, USA) with the usual cementless THA surgical technique. To change the position of the pelvis three‐dimensionally as desired, the hemi‐pelvis was held using a free camera platform and a tripod (SLIK, Saitama, Japan). Using a strong bond (SU; Konishi, Osaka, Japan), a pedestal of the hemi‐pelvis was attached to a square acrylic plate (900 cm^2^; thickness, 5 mm) so that the longer edge of the pedestal was parallel to the plate. The center of rotation of the hip joint was projected onto the acrylic plate, and a 1/4‐inch female screw was placed. Then, a male screw of the free camera platform was tightly twisted into the female screw. A #12 femoral broach of the Synergy Select™ hip system (Asian version of Synergy, Smith & Nephew, Memphis, TN, USA) was tightly seated into a Sawbone femur (model 1121, Sawbones, Vashon Island, WA, USA) using the usual femoral preparation. A high‐offset trial neck with a 28‐mm diameter and cobalt‐chromium femoral head (+ 0 neck length) was attached to the femoral broach. A bar was fixed to the hole at the shoulder of the broach as an indicator of rotation. The bar was perpendicular to the long axis of the neck. The Sawbone model of the femur was held using a custom‐made stand. Finally, the stand was fixed to a solid metal base board (width, 50 cm; length, 90 cm; and thickness, 2 cm) in the neutral femoral position (the long axis of the femur was horizontal and parallel to the longer edge of the base board, and the bar was horizontal). Two reference pins were placed in the ilium. The first pin was placed in the iliac tubercle (P1), and the second was placed at the top of the iliac crest, intersecting the line of the long axis of the femur when the pelvis was in the neutral position (P2). The pins were set perpendicular to the acrylic plate. The lateral prominence of the greater trochanter was chosen as the measurement point. A 3.5‐mm cortical screw was placed to mark the point clearly.

### Definition of Rotatory Directions

**Fig. 3:**
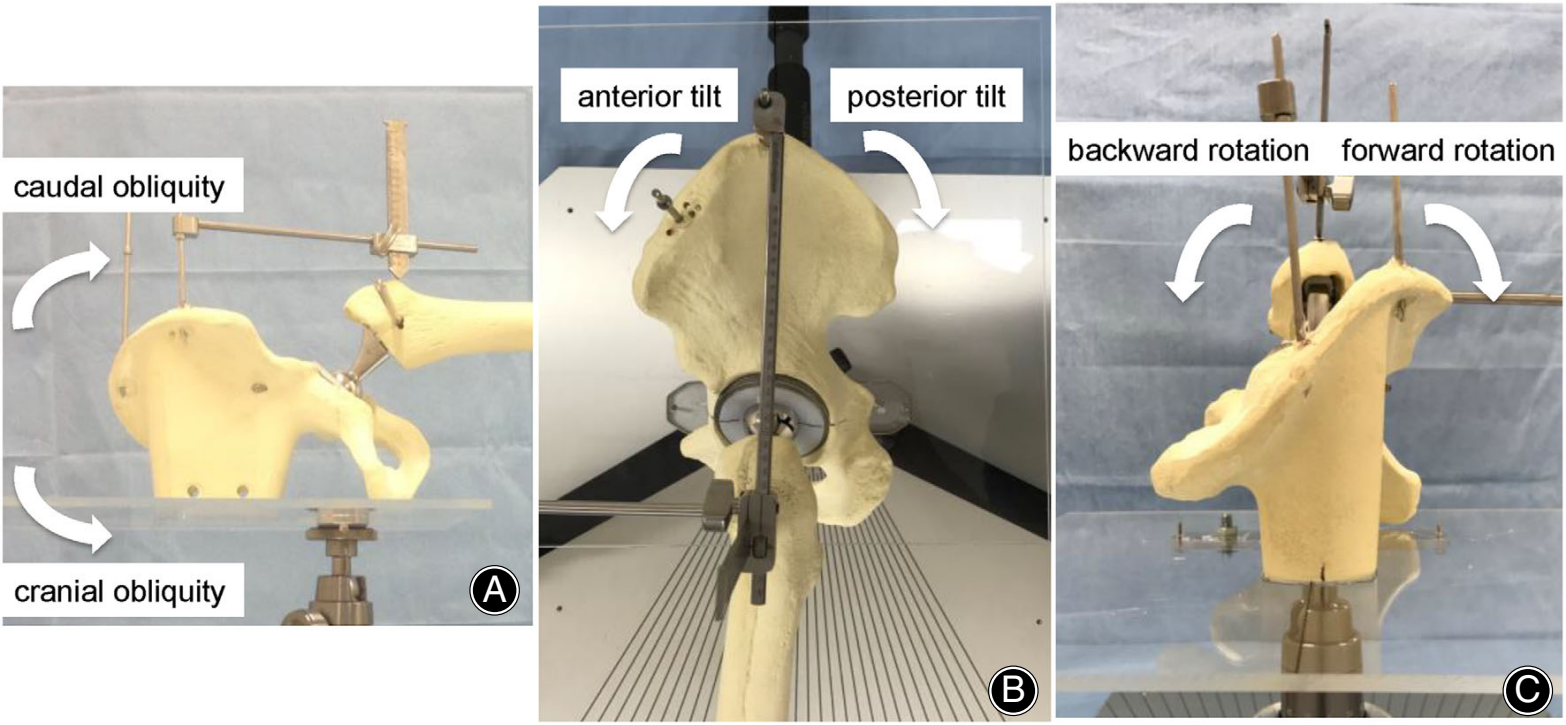
Definition of pelvic movements. (A) Obliquity is a pelvic movement around the antero‐posterior axis (coronal plane) with cranial and caudal obliquity. (B) Tilt is a pelvic movement around the transverse axis (sagittal plane), with anterior and posterior tilts. (C) Rotation is a pelvic movement around the longitudinal axis (axial plane), with forward and backward rotations

The position of the pelvis was divided into three rotatory directions according to the definition of Grammatopoulos *et al*., namely, obliquity, tilt, and rotation (Fig. 3).^19^ Obliquity is a pelvic movement around the antero‐posterior axis (coronal plane), with cranial and caudal obliquity corresponding to femoral adduction and abduction, respectively. Tilt is a pelvic movement around the transverse axis (sagittal plane), with anterior and posterior tilts corresponding to femoral flexion and extension, respectively. Rotation is a pelvic movement around the longitudinal axis (axial plane), with forward and backward rotations corresponding to femoral external and internal rotations, respectively. When the acrylic plate was horizontal, obliquity and rotation were defined as 0°. When the four sides of the acrylic plate were parallel to those of the metal baseboard, the tilt was defined as 0°. The neutral position of the pelvis was defined as a 0° obliquity, tilt, and rotation.

### Measurement of LL and OS Errors

To determine LL and OS measurement errors associated with pelvic position, the pelvic position was changed in one rotatory direction while the other rotatory directions were fixed. The range of obliquity was from 10° of cranial obliquity to 10° of caudal obliquity in 2° increments. The range of tilt was from 30° of anterior tilt to 30° of posterior tilt in 5° increments with reference to the scale of the free camera platform. The range of pelvic rotation was from 16° of forward rotation to 16° of backward rotation in 2° increments. For obliquity and rotation, the angle of the acrylic plate for each position was measured using a digital angle gauge (WR 300; Wixey, FL, USA). The LOMI was set to P1 or P2. LL and OS were measured three times for each pelvic position, and the mean values were calculated. The differences in the values of LL and OS between neutral and each pelvic position were defined as errors. Error of the pelvic position in one rotation is shown by line graphs. The line graphs were prepared using Microsoft Excel for Microsoft Office365 (Microsoft Corp, Redmond, WA, USA).

## Results

### Analysis of Pelvic Obliquity

**Fig. 4:**
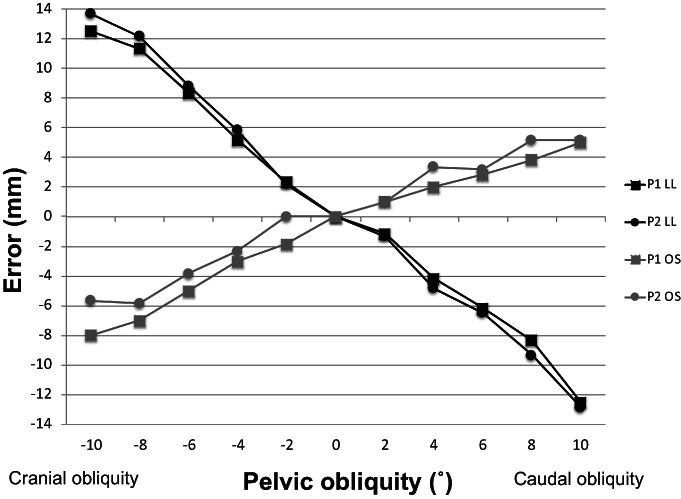
Leg length and offset measurement errors associated with pelvic obliquity. LL: leg length, OS: offset

Pelvic obliquity had an enormous effect on the LL, irrespective of the pin position (Fig. 4). In both P1 and P2, the LL became longer in cranial obliquity and shorter in caudal obliquity; the total error was 25.0 mm for P1 and 26.5 mm for P2. Similarly, pelvic obliquity had a significant effect on the OS, irrespective of the pin position. In both P1 and P2, the OS became smaller in cranial obliquity and larger in caudal obliquity; the total error was 13.0 mm for P1 and 10.9 mm for P2. A cranial or caudal obliquity of only 4° caused an error of approximately 5 mm in the LL and 3 mm in the OS.

### Analysis of Pelvic Tilt

**Fig. 5:**
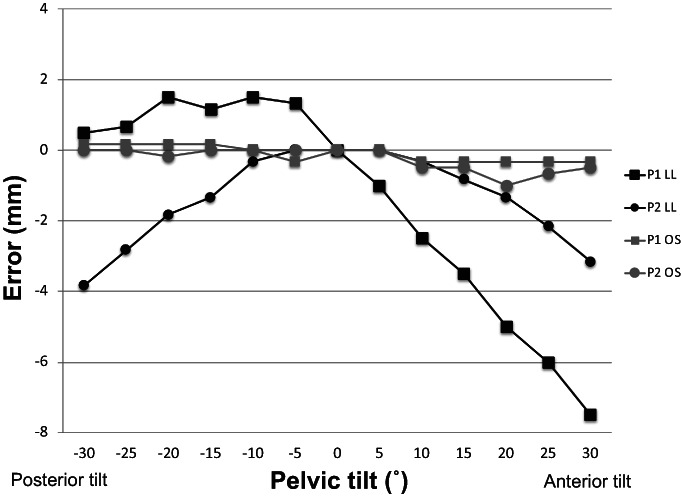
Leg length and offset measurement errors associated with pelvic tilt. LL: leg length, OS: offset

The effect of the pelvic tilt on the LL was different for P1 and P2 (Fig. 5). For P1, the LL became shorter in anterior tilt and slightly longer in posterior tilt; the error was 9.0 mm in total. For P2, the LL became shorter in both anterior and posterior tilts; the total error was 3.8 mm. The pelvic tilt had an extremely small effect on OS, within 1.0 mm for both P1 and P2.

### Analysis of Pelvic Rotation

**Fig. 6:**
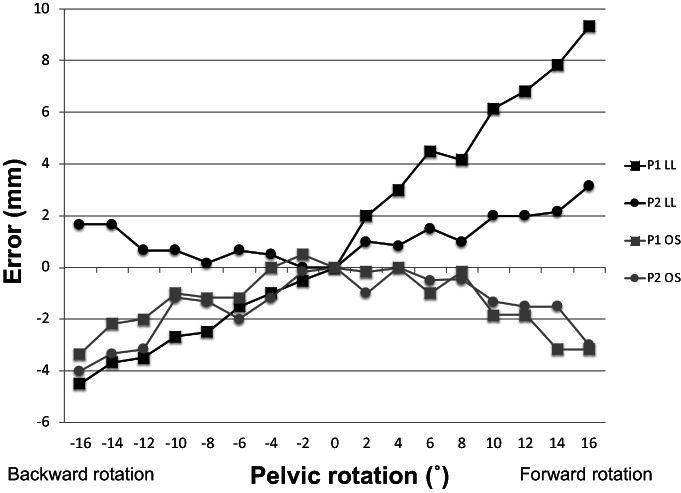
Leg length and offset measurement errors associated with pelvic rotation. LL: leg length, OS: offset

The effect of pelvic rotation on the LL was also different between P1 and P2 (Fig. 6). For P1, the LL became longer in forward rotation, whereas it became shorter in backward rotation; the error was 13.8 mm in total. For P2, the LL became longer in both forward and backward rotations; the error was 3.2 mm in total. The effect of pelvic rotation on OS was similar for P1 and P2. OS became smaller with increases in forward and backward rotation; the error was 3.8 mm for P1 and 4.0 mm for P2.

## Discussion

This study revealed important findings. First, pelvic obliquity had a profound effect on LL and OS errors, irrespective of the pelvic reference position. Second, we showed that for pelvic tilt and rotation, the LL error was smaller for the reference on the top of the iliac crest than that for the iliac tubercle.

### Impact of Positional Changes on LL and OS Measurements

Overall, the results of this study are consistent with those of previous femoral movement studies.^13^, ^14^ First, pelvic obliquity, which corresponds to hip abduction and adduction, had a profound effect on LL and OS errors, irrespective of the pelvic pin position. A pelvic obliquity of 20° resulted in an error of approximately 25 mm in the LL and 10.9–13 mm in the OS. In our previous study, LL and OS errors were approximately 24 mm and 13 mm, respectively, from 10° of adduction to 10° of abduction.^14^ Under different experimental conditions, Sarin *et al*. found that 10° of hip abduction or adduction resulted in a 14–17 mm error for the LL and a 9.5–11.1 mm error for the OS.^13^ Collectively, these results show that both pelvic obliquity and femoral abduction/adduction greatly affect the LL and OS. Therefore, not only the femoral position, but also the pelvic position should be strictly maintained during LL and OS measurements in THA.

### Appropriate Pelvic Reference Pin Position

In the current study, the LL error for the pelvic reference in line with the long axis of the femur (P2) was smaller than that for the pelvic reference in the iliac tubercle (P1) for pelvic tilt and rotation. As for the pelvic tilt, the total LL error from 30° of anterior tilt to 30° of posterior tilt was smaller in P2 (3.8 mm) than that in P1 (9.0 mm). As for the pelvic rotation, the total LL error from 16° of forward rotation to 16° of backward rotation was much smaller in P2 (3.2 mm) than that in P1 (13.8 mm). These findings were in agreement with those of a previous report.^14^ Therefore, the pelvic reference in line with the long axis of the femur when the pelvis is in the neutral position (namely, P2) should be chosen as the reference pin position in the ilium. Theoretically, no OS error should be observed because of the pelvic tilt because the tilt is movement in the sagittal plane. In this study, we observed an OS error due to a pelvic tilt of <1 mm. The result supports that the present measurements are fairly accurate. The effects of pelvic rotation on the OS were smaller than those on the LL. Moreover, the observed OS error was almost similar irrespective of the position of the pelvis pin.

### Clinical Relevance of LL and OS Measurement Errors

During actual THA, modular heads are used to adjust the LL and OS. Given that the neck‐shaft angles of the femoral stems are approximately 130°, a 3‐mm neck‐length change brings about an LL and OS change of approximately 2 mm. Therefore, a measurement error of less than 2 mm in the LL and OS is acceptable.^14^ Table 1 shows the acceptable ranges of pelvic obliquity, tilt, and rotation according to the requirements. The acceptable range of pelvic obliquity for the LL and OS is extremely narrow, regardless of the pelvic reference. For pelvic tilt, the acceptable range in the LL is comparable to that in P1 (35°) and P2 (40°), although only 5° of anterior tilt is acceptable in P1. For pelvic rotation, the acceptable range in the LL is narrower in P1 and wider in P2, whereas the acceptable range in OS is wider, regardless of the pelvic reference. From the viewpoint of the acceptable range of LL error, P2 provides a relatively wide range of pelvic tilt and rotation. Asayama *et al*.^20^ measured intraoperative pelvic motion in THA, using a posterolateral approach and reported that the ranges of pelvic obliquity, tilt, and rotation ranged from 6° of cranial obliquity to 9° of caudal obliquity, from 18° of anterior tilt to 10° of posterior tilt, and from 5° to 31° of forward rotation. Their results showed that LL and OS measurement errors could occur in actual THA. Furthermore, when using a portable navigation system, the accuracy of acetabular component placement has been reported to reduce with pelvic positional change in all three rotatory directions.^21^ Therefore, minimizing pelvic positional change is desirable. One method for reducing pelvic motion is using the hip positioner, which fixes both the anterior superior iliac spine and sacrum as well as contributes to intraoperative pelvic stability.^16^ Hence, pelvic positional change should be carefully managed.

**TABLE 1:** Acceptable range (error <2 mm) of pelvic obliquity, tilt, and rotation^a^

		Leg length		Offset	
		P1	P2	P1	P2
Obliquity	Cranial	0	0	2	2
	Caudal	2	2	2	2
Tilt	Anterior	5	20	30	30
	Posterior	30	20	30	30
Rotation	Forward	0	8	12	14
	Backward	6	16	12	10

^a^ Values are presented as degrees.

*Note*: Positions where the pin is placed: P1, the iliac tubercle; P2, the top of the iliac crest intersecting the line of the long axis of the femur when the pelvis is in the neutral position

### Strengths and Limitations

To the best of our knowledge, this is the first study to quantitatively show that pelvic positional changes have an effect on LL and OS measurement errors. In previous experimental studies, the position of the pelvis was fixed, while the position of the femur was changed during LL and OS measurements.^13^, ^14^ In contrast, in the present study, the position of the femur was fixed while the position of the pelvis was changed. From the viewpoint of the relative movement between the pelvis and the femur, either experimental setting might yield similar results, although this has not been proven to date.

This study had some limitations. First, this result is probably limited to the experimental model of this study. In clinical practice, bone and implant sizes vary and the absolute value of measurement error may also vary. Second, although the pelvis could move in combination with obliquity, tilt, and rotation during an actual THA, the errors of LL and OS in the case of combined pelvic movement were not investigated. Third, not only the pelvis, but also the femur can move during the actual THA.^6^, ^15^ The combined movement of the pelvis and femur could either increase or decrease the errors in LL and OS measurements.

### Conclusion

In conclusion, pelvic obliquity has an enormous effect on LL and OS measurement errors, regardless of the reference pin position. Given the small LL measurement error for pelvic tilt and rotation, the pelvic reference in line with the long axis of the femur when the pelvis is in the neutral position should be chosen as the reference pin position in the ilium.

## Author Contributions

All authors had full access to the data in the study and take responsibility for the integrity of the data and the accuracy of the data analysis. *Conceptualization*, H.K.; *Methodology*, N.K. and H.K.; *Investigation*, N.K. and H.K.; *Formal Analysis*, N.K. and H.K.; *Resources*, H.K.; *Writing ‐ Original Draft*, N.K.; *Writing ‐ Review & Editing*, H.K. and T.N.; *Visualization*, J.D.B.; *Supervision*, H.M.

## Authorship Declaration

All authors listed meet the authorship criteria according to the latest guidelines of the International Committee of Medical Journal Editors, and all authors are in agreement with the manuscript.

## Disclosure Statement

The authors declare that there is no conflict of interest.

## References

[1] Li J , McWilliams AB , Jin Z , et al. Unilateral total hip replacement patients with symptomatic leg length inequality have abnormal hip biomechanics during walking. Clin Biomech. 2015;30:513–9. 10.1016/j.clinbiomech.2015.02.014 PMC4441097 25900447

[2] Sakalkale DP , Sharkey PF , Eng K , Hozack WJ , Rothman RH . Effect of femoral component offset on polyethylene wear in total hip arthroplasty. Clin Orthop Relat Res. 2001;388:125–34. 10.1097/00003086-200107000-00019 11451111

[3] Upadhyay A , York S , Macaulay W , McGrory B , Robbennolt J , Bal BS . Medical malpractice in hip and knee arthroplasty. J Arthroplasty. 2007;22:2–7. 10.1016/j.arth.2007.05.003 17823005

[4] McWilliams AB , Douglas SL , Redmond AC , et al. Litigation after hip and knee replacement in the National Health Service. Bone Jt J. 2013;95:122–6. 10.1302/0301-620X.95B1.30908 23307685

[5] Röder C , Vogel R , Burri L , Dietrich D , Staub LP . Total hip arthroplasty: leg length inequality impairs functional outcomes and patient satisfaction. BMC Musculoskelet Disord. 2012;13:95. 22686325 10.1186/1471-2474-13-95 PMC3495212

[6] Ranawat CS , Rao RR , Rodriguez JA , Bhende HS . Correction of limb‐length inequality during total hip arthroplasty. J Arthroplasty. 2001;16:715–20. 11547369 10.1054/arth.2001.24442

[7] Bourne RB , Rorabeck CH . Soft tissue balancing: the hip. J Arthroplasty. 2002;17:17–22. 12068397 10.1054/arth.2002.33263

[8] Renkawitz T , Weber T , Dullien S , Woerner M , Dendorfer S , Grifka J , et al. Leg length and offset differences above 5 mm after total hip arthroplasty are associated with altered gait kinematics. Gait Posture. 2016;49:196–201. 27450670 10.1016/j.gaitpost.2016.07.011

[9] Mahmood SS , Mukka SS , Crnalic S , Wretenberg P , Sayed‐Noor AS . Association between changes in global femoral offset after total hip arthroplasty and function, quality of life, and abductor muscle strength. A prospective cohort study of 222 patients. Acta Orthop. 2016;87:36–41. 26471772 10.3109/17453674.2015.1091955 PMC4940589

[10] Sariali E , Klouche S , Mouttet A , Pascal‐Moussellard H . The effect of femoral offset modification on gait after total hip arthroplasty. Acta Orthop. 2014;85:123–7. 24564749 10.3109/17453674.2014.889980 PMC3967252

[11] Barbier O , Ollat D , Versier G . Interest of an intraoperative limb‐length and offset measurement device in total hip arthroplasty. Orthop Traumatol Surg Res. 2012;98:398–404. 22560790 10.1016/j.otsr.2012.02.004

[12] Fansur M , Yurdi NA , Stoewe R . The intraoperative use of a calliper predicts leg length and offset after total hip arthroplasty. Component subsidence influences the leg length. J Orthop Surg Res. 2021;16:424. 34217347 10.1186/s13018-021-02559-3 PMC8254249

[13] Sarin VK , Pratt WR , Bradley GW . Accurate femur repositioning is critical during intraoperative total hip arthroplasty length and offset assessment. J Arthroplasty. 2005;20:887–91. 16230240 10.1016/j.arth.2004.07.001

[14] Kawamura H , Watanabe Y , Nishino T , Mishima H . Effects of lower limb and pelvic pin positions on leg length and offset measurement errors in experimental total hip arthroplasty. J Orthop Surg Res. 2021;16:193. 33726774 10.1186/s13018-021-02347-z PMC7962310

[15] Zhu J , Wan Z , Dorr LD . Quantification of pelvic tilt in total hip arthroplasty. Clin Orthop Relat Res. 2010;468:571–5. 19714387 10.1007/s11999-009-1064-7 PMC2806995

[16] Mittal A , Chetty N , Pham T , Shah I , Raji R , Leasure J , et al. Pelvic stability during simulated total hip arthroplasty motions: comparing different hip positioners. J Orthop. 2022;34:398–403. 36325517 10.1016/j.jor.2022.08.004 PMC9618677

[17] Kamenaga T , Hayashi S , Hashimoto S , et al. Intraoperative pelvic movement is associated with the body mass index in patients undergoing total hip arthroplasty in the supine position. J Orthop Sci. 2020;3:446–51. 10.1016/j.jos.2019.05.010 31174965

[18] Ezoe M , Naito M , Asayama I , Ishiko T , Fujisawa M . Pelvic motion during total hip arthroplasty with translateral and posterolateral approaches. J Orthop Sci. 2005;10:167–72. 15815864 10.1007/s00776-004-0880-6

[19] Grammatopoulos G , Pandit HG , da Assunção R , et al. Pelvic position and movement during hip replacement. Bone Jt J. 2014;96–B:876–83. 10.1302/0301-620X.96B7.32107 24986939

[20] Asayama I , Akiyoshi Y , Naito M , Ezoe M . Intraoperative pelvic motion in total hip arthroplasty. J Arthroplasty. 2004;19:992–7. 15586335 10.1016/j.arth.2004.03.013

[21] Asai H , Takegami Y , Seki T , Ishiguro N . Pelvic tilt reduces the accuracy of. Acetabular component placement when using a portable navigation system: an in vitro study. Arthroplasty Today. 2021;7:177–81. 33553546 10.1016/j.artd.2020.12.012 PMC7856392

